# Soluble Milk Protein Supplementation with Moderate Physical Activity Improves Locomotion Function in Aging Rats

**DOI:** 10.1371/journal.pone.0167707

**Published:** 2016-12-14

**Authors:** Aude Lafoux, Charlotte Baudry, Cécile Bonhomme, Pascale Le Ruyet, Corinne Huchet

**Affiliations:** 1 INSERM U1087 Institut du thorax, Therassay, Université de Nantes, Nantes, France; 2 LACTALIS Recherche et Développement, Retiers, France; 3 LACTALIS Nutrition Santé, Torce, France; University of Birmingham, UNITED KINGDOM

## Abstract

Aging is associated with a loss of muscle mass and functional capacity. Present study was designed to compare the impact of specific dairy proteins on muscular function with or without a low-intensity physical activity program on a treadmill in an aged rat model. We investigated the effects of nutritional supplementation, five days a week over a 2-month period with a slow digestible protein, casein or fast digestible proteins, whey or soluble milk protein, on strength and locomotor parameters in sedentary or active aged Wistar RjHan rats (17–19 months of age). An extensive gait analysis was performed before and after protein supplementation. After two months of protein administration and activity program, muscle force was evaluated using a grip test, spontaneous activity using an open-field and muscular mass by specific muscle sampling. When aged rats were supplemented with proteins without exercise, only minor effects of different diets on muscle mass and locomotion were observed: higher muscle mass in the casein group and improvement of stride frequencies with soluble milk protein. By contrast, supplementation with soluble milk protein just after physical activity was more effective at improving overall skeletal muscle function in old rats compared to casein. For active old rats supplemented with soluble milk protein, an increase in locomotor activity in the open field and an enhancement of static and dynamic gait parameters compared to active groups supplemented with casein or whey were observed without any differences in muscle mass and forelimb strength. These results suggest that consumption of soluble milk protein as a bolus immediately after a low intensity physical activity may be a suitable nutritional intervention to prevent decline in locomotion in aged rats and strengthen the interest to analyze the longitudinal aspect of locomotion in aged rodents.

## Introduction

Sarcopenia is the involuntary decline in lean muscle mass, strength, and function that occurs with aging [1–3]. It is generally thought that the risk of disability and loss of functional capacity in the elderly is increased by sarcopenia. Multiple interrelated processes lead to the development of sarcopenia. These include hormonal, metabolic, immunological, neurological, as well as nutritional factors [4, 5]. In older adults, inadequate protein intake is also often cited as being strongly correlated with lower muscle mass [6, 7] physical performance, and muscle strength [8]. Furthermore, several studies have demonstrated beneficial effects of dietary protein supplementation in the elderly [9–11]. There is increasing evidence that physical training can counteract age-related muscle loss and functional decline, even in frail elderly adults [12–17]. These findings support the notion that reduced physical activity is implicated in the aetiology of age-related decline in muscle mass [18, 19], although it must be kept in mind that these two age-related impairments may reciprocally be cause and effect for one another. If increased levels of physical activity and aerobic fitness are associated with lower risk of cardiovascular morbidity and mortality in elderly subjects, it has been proposed that modest low physical activity as around 7000–8000 steps per day may protect against sarcopenia [20, 21]. Physical inactivity and inadequate dietary protein intake seem to largely contribute to age-related muscle loss, impaired function, and disability. Combining exercise and adequate protein intake, which has been reported in recent years to be a key component for prevention and management of sarcopenia [22], may hence provide a synergistic and incremental effect on skeletal muscle mass and capacities. Studies in elderly individuals have nonetheless shown that protein supplementation (with a total protein intake of twice the recommended dietary allowance) in combination to physical exercise did not further increase muscle mass, strength, and/or muscle protein synthesis compared to levels achieved with exercise alone [23, 24]. However, one randomized control trial (RCT) has demonstrated that an increase in dietary protein (*i*.*e*. 30 g/day/week during 24 weeks) combined with twice-weekly progressive resistance training in a frail elderly population induced an average increase of 1.3 kg in lean body mass compared with exercise alone, although there was no effect of protein intake on strength or physical performance, with both the supplemented and the un-supplemented group experiencing a similar degree of improvement [16]. More recently, another RCT in non-frail elderly subjects who underwent resistance training has reported a significant additional beneficial effect on muscle strength of a cysteine-rich whey *versus* casein protein supplementation [17]. Volek et al. have also described the greater effectiveness of supplementation with whey *versus* soy protein in increasing leucine plasma concentrations and promoting gains in lean body mass in trained adults [25]. These studies have underscored the importance of the nature of the proteins selected for the nutritional strategy, and particularly their digestibility rate (*i*.*e*. rapidly versus slowly digested proteins) [26], as well as their amino-acid composition [27].

To our knowledge, the potential effects of a supplementation with specific proteins on the muscular benefits derived from physical activity have not been thoroughly examined in a rodent model of aging. This study was hence undertaken to comprehensively compare the effects of different types of dairy protein supplements on gait properties in old rats in sedentary condition or submitted to a low-intensity exercise program. Thus, to investigate locomotion properties of the aged rats, pre- and post-measurements of dynamic/spatial parameters of unforced walking were obtained with an automated quantitative gait analysis system. 17-month-old Wistar rats were supplemented over a 2-month period with boluses of various proteins extracted from milk (*i*.*e*. casein CAS, a slowly digested protein; soluble milk protein PRO, a rapidly digested protein) or from cheese manufacture (*i*.*e* whey protein WHEY, a rapidly digested protein) with and without a concomitant exercise program on a treadmill. At the end of protocol period, spontaneous activity, muscle strength and mass were also analysed.

## Materials and Methods

### Animals

This study was approved by the Pays de la Loire (France) Ethics Committee for Animal Experimentation, and it was in accordance with the guidelines of the French National Research Council for the Care and Use of Laboratory Animals (Permit Numbers: CEEA-PdL-01579.01). All reasonable efforts were made to minimize animal suffering during the study.

48 aged male Wistar rats (17-months-old) obtained from Janvier Labs (Laval, France) and weighing 495±5 g on average were used in the following behavioural studies. Rats have finer and more accurate motor coordination than mice and exhibit a richer behavioural display, including more complex social traits that allows a better manipulation for the training procedure on treadmill [28]. The 17-to 18 month-old rats were regarded as old rats while 22-24-month-old rats were considered as senescent [29]. Seventeen-month-old Wistar rats were chosen and supplemented during 2 months as these ages preceded the increase of mortality seen in the older age rats. Furthermore, Wistar rat is an established model system for studying skeletal muscle impairment as sarcopenia [30]. The age-related changes described in human muscle, the loss of fast motor units, muscle atrophy, conversion of fast-twitch to slow-twitch fibers and decrease in force were also observed in this rat model. Animals were housed individually in a controlled environment (*i*.*e*. an ambient temperature of 21±1°C, 12-h light/dark inverted cycle), maintained on a low-protein (10%) standard diet (SAFE A04, SAFE, Augy, France) and with *ad libitum* water, and they were handled daily for several weeks prior to the experimentation. Food consumption and animal weights were monitored weekly.

### Stimulus exercise

Animals were randomly assigned to 3 groups according to protein supplements and balanced for body weights (BW) as CAS (casein) group, average BW of 493±8 g, *n = 16*, WHEY group, average BW of 495±8 g, *n = 16*, and PRO (soluble milk protein) group, average BW of 497±10 g, *n = 16*. Half of each treatment group was assigned to a concurrent treadmill activity program. At the beginning of the dark (active) phase, rats were introduced to the treadmill, which had a Plexiglas^®^ cover to prevent their escape, and which provided a running surface that was 100 cm in length and 15 cm in width. Initial 10-min physical activity sessions of slow speed walking (5 m/min) were performed to familiarize the animals with the equipment. During the first two weeks, the speed of the treadmill and the duration of the session were progressively increased so that the old rats were able to walk for 30 min at a speed of 10 m/min without enforcement by an electrical shock. This moderate exercise program was very well tolerated by all the animals and they were classified as “active old rats”. The other half of the rat population was introduced for the same duration to the immovable treadmill, and they were classified as “sedentary old rats”. This protocol was maintained over a 2-month period, until the rats reached the age of 19 months.

### Protein supplementation

During 8 weeks, protein supplements were administered once a day, 5 days a week, in the form of a 12 mL bottled free access drinking solution (*i*.*e*. a bolus) which contained either 0.85 g of casein (for the CAS group), whey protein (for the WHEY group), or soluble milk protein (for the PRO group). Casein (CAS) is a slowly digested protein while whey (WHEY) and soluble milk protein (PRO) are rapidly digested proteins. Comparison between the soluble milk protein and whey was made due to difference between their production process and amino acids composition. Indeed, soluble milk protein is made directly from milk and not from whey as is usual with a low temperature process. Protein composition of soluble milk protein are native and the amino acid composition as illustrated in the Table 1 demonstrated that soluble milk protein contains more leucine. Protein supplementation *i*.*e* boluses were given to the rats immediately after the treadmill session for the active and just after a same period of time in an immobilized treadmill for the sedentary group. Three hours after the beginning of the dark (active) phase all rats were supplemented with proteins. Protein solution was generally consumed within 20 min and was considered by rat as a reward. This procedure allowed us to avoid electrical shocks during the exercise activity. The boluses also contained 0.2% sucrose to increase appetence, and thus allowed assimilation over a short-time period without causing drinking water privation. As for exercise stimulus, this protein supplementation was maintained over a 2-month period, until the rats reached the age of 19 months. Proteins were all provided by Lactalis Ingredients, Bourgbarré France and their amino acids compositions are displayed in Table 1.

**Table 1:** Amino acids composition of proteins.

	CASEIN (for CAS group)	WHEY (for WHEY group)	PROLACTA (for PRO group)
Leucine	10,0	11,2	12,6
Isoleucine	5,6	6,8	5,9
Valine	6,9	6,2	5,8
Tryptophan	1,3	2,0	2,5
Thréonine	4,6	8,0	5,4
Lysine	8,4	9,2	9,9
Phénylalanine	5,4	3,5	4,3
Histidine	3,0	1,9	2,2
Méthionine	3,0	4,7	5,1
Cystine	0,7	2,5	2,7
Aspartic acid	7,4	11,3	11,5
Glutamic acid	22,8	18,6	18,7
Alanine	3,1	5,2	4,8
Arginine	4,0	2,8	3,3
Glycine	1,9	2,0	2,1
Proline	11,2	6,4	5,4
Sérine	5,9	5,9	5,3
Tyrosine	6,4	3,4	4,4

Amino acids (g) per 100 g of protein

### Musculoskeletal function

**Fig 1:**
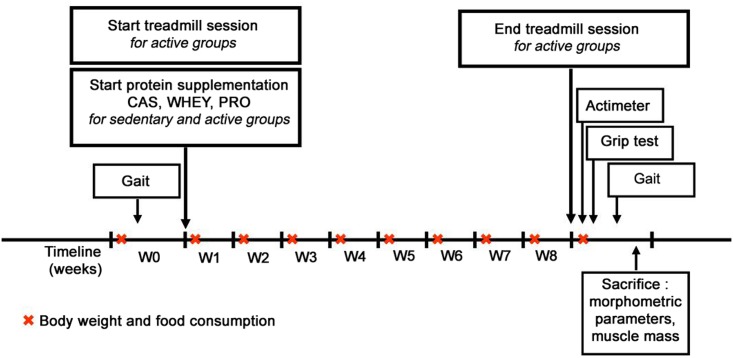
Schematic study design. Study design and time line for the experimental protocol for all old rats over the 2-month period of study. W0 to W8: weeks of protocol.

As illustrated in Fig 1, *in vivo* tests were performed in the same sequence for each rat, with equivalent time of rests between the tests. To avoid bias in the analysis, all experiments were done in a blind manner. The main functional objective of this study was to analyze locomotion by using the gait system. In this context, this methodology was investigated before and after supplementation and treadmill session. But in order to explore muscle function, grip strenght and locomotion were also investigated at the end point. Actimeter and grip test could not be easily investigated in a longitudinal aspect. Indeed, grip test is a stress-inducing method in aged rats and, in order to limit stress and a possible death of the rats, measurements of the strength were performed only at the end of the study *i*.*e*. after 8 weeks of supplementation and physical activity program. Actimeter is often used to investigate locomotion in rodents but a pronounced habituation exhibited as a decrease in locomotion was observed after several trials. By contrast, with the gait experiments performed before and after 2 months of supplementation, there is limited stress and rats were encouraged to move freely across the walkway of the GaitLab system. This last point is one of the elements strengthening the use of the GaitLab system instead of the actimeter to analyze locomotion in a longitudinal aspect in aged rodents.

#### Gait analysis

For all the rats in the experimental protocol, gait parameters for unforced walking rats were analyzed before and after the 2-month period of protein supplementation using the GaitLab system (ViewPoint) (Fig 1). The GaitLab system consisted of a 2 m long glass walkway plate, illuminated with green light that is reflected within the glass at touched points, a high-speed video camera, and a software package for quantitative assessment of animal footprints. This system was used to analyze the gait of unforced moving rats, as described below. The rats were encouraged to move across the walkway, which was located in a dedicated soundproof room, over four (prior to treatment) and three (following treatment) consecutive days, and for at most five times per day. Neither food deprivations, nor food rewards, were used as motivators, but a goal box (*i*.*e*. the homecage) was located at one end of the walkway. In an effort to capture the range of preferred speeds and the best performances, we strived to capture as many successful ‘free-ranging’ trials as possible, in order to acquire a range of speeds. A successful run was defined when an animal finished running the tracks without any interruption or hesitation, with regularity calculated by associated software up to 98% of the trial. Out of the 48 rats tested, one old rat in the PRO sedentary group, and another one in the WHEY active group, failed to run without any interruption. These two rats were hence excluded from this gait assessment.

For each successful trial, gait parameters were generated from a sequence consisting of at least 3 interrupted strides per paw, and included both temporal and spatial measurements. Table 2 lists the definitions of the gait parameters used in this study. The time lags (fore, hind, left, and right lags) were used in order to identify gait use by the animals [31]. Briefly, analysis of the time lags between the two feet of the pars (*i*.*e*. fore lag and hind lag) allows symmetrical and asymmetrical gaits to be distinguished according to the model of Hildebrand [32, 33]. By definition, for symmetrical gaits these time lags are the same and equal to 50% of the cycle duration. By contrast, any gait where hindlimbs or forelimbs fell either as more or as less than 50±5% of the stride cycle were treated as being asymmetrical. The successions of the movements are the result of the time lag between the action of ipsilateral fore and hind paw (left and right lag). Analysis of these time lags then allowed us to identify the various types of symmetrical (*i*.*e*. trot, diagonal, or lateral walk) and asymmetrical (*i*.*e*. transverse or rotary gallop) walking used by the rats [31]. Other temporal and spatial gait parameters were determined for each paw, and expressed as averages of the data obtained on at least 3 consecutive strides by the limbs for each successful trial.

**Table 2:** Definition of gait parameters.

Gait parameters	Definition
Fore lag	Time lag between forefeet footfall expressed as a percentage of the stride time
Hind lag	Time lag between hindfeet footfall expressed as a percentage of the stride time
Left lag	Time lag between left feet footfall expressed as a percentage of the stride time
Right lag	Time lag between right feet footfall expressed as a percentage of the stride time
Stride time	Time lag in seconds between two consecutive initial contacts by the same paw
Stride frequency	Number of gait signals over time (number of strides per minute)
Stance time	Duration in seconds of contact of a paw with the walking surface
Duty factor	Expression of the stance time as a percentage of the stride time
Brake time	Duration in seconds of increasing paw contact area over time during the stance time
Stride length	Distance between successive placements of the same paw

Temporal and spatial measures were generated from a sequence consisting of at least 3 interrupted strides per paw selected from any of the successful trials. The latter being defined as having occurred when an animal finishes running the tracks without any interruption or hesitation, with a regularity of up to 98%.

#### Short term-spontaneous activity

At the end of the week 8, motor behaviour was examined in an open field actimeter for all rats (Fig 1) [34]. For this analysis, rats were individually placed in an automated photocell activity chamber (Letica model LE 8811, Bioseb, France) which consisted of a Plexiglas^®^ chamber (dimensions of 45 cm×45 cm×50 cm) surrounded by two rows of infrared photobeams. The first row of sensors was positioned at a height of 7 cm for measuring horizontal activity, and the second row was positioned above the animals to measure vertical activity. Spontaneous motor activity was measured over a period of 5 min using a movement analysis system (Bioseb, France), which dissociates the activity time (s), the total number of movements (nb), and the total distance travelled (cm). The average speed (cm/s) was also calculated by dividing the total distance travelled by the activity time.

#### Grip strength

As for the open-field actimeter at the end of the week 8, in order to see whether protein treatment could affect skeletal muscle strength, all rats were challenged using the grip test (Fig 1). Rats were placed with their forepaws on a T-bar, and they were gently pulled backwards until they released their grip [35]. A grip meter (Bio-GT3, BIOSEB, France), attached to a force transducer, measured the peak force generated. Five tests were performed sequentially. The results are expressed as the mean of 3 median values in grams (g), and normalized by the body weight (g/g).

#### Morphometric parameters

At the end of the protocol, animals were anesthetized with a mixture of ketamine (100 mg/kg, Imalgene, Merial, Lyon, France) and xylazine (10 mg/kg, Rompun, Bayer, Leverkusen, Germany) (Fig 1). Rats were then sacrificed by intravenous administration of sodium pentobarbital (300 mg, Dolethal, Vetoquinol UK Ltd, Buckingham, UK). Immediately after being sacrificed, the body weight (g) and the body length (cm) of each rat were measured to determine the body mass index, which was calculated as body weight/(body length)^2^ (g/cm^2^*)*. The *tibialis anterior* (TA), *extensor digitorum longus* (Edl) and *soleus* muscles of the hindlimb, and the *biceps brachii* (BB) and *extensor digitorum carpi* (Edc) muscles of the forelimb were sampled and weighed.

### Statistical analyses

Statistical evaluation of the data was performed by the non-parametric Kruskal-Wallis test in order to analyse the difference between protein supplements under the two conditions, *i*.*e*. sedentary or active rats. When a significant overall effect was detected, differences across sedentary or active groups were assessed by Dunn’s post hoc test. For gait parameters a Wilcoxon matched-paired signed rank test was performed. Additionally, weekly measurement (e.g. body weight, food intake) were analyse by Friedman test following by Dunn’s post hoc test. All analyses were performed using GraphPad Prism 5 (GraphPad Software, Inc., La Jolla, CA, USA). The data are presented as the mean ± SEM, with the significance level set at p < 0.05.

## Results

### Equal effectiveness of the different types of proteins to increase protein intake

For each group, body weights were similar at the beginning of the study (Table 3). Body weights increased significantly over the course of the 2-month study period. However this weight gain was similar regardless of the type of protein for both the sedentary (CAS: +8.0±1.3%, n = 8, WHEY: +8.9±1.1%, n = 8, PRO: +9.9±0.8%, n = 8) and the active group (CAS: +5.6±1.8%, n = 8; WHEY: +5.3±1.9%, n = 8; PRO: +5.0±2.1%, n = 8).

**Table 3:** Similar body weights and final morphometric parameters after protein supplementation in sedentary or active old rats.

	Sedentary old rats			Active old rats		
Parameters	CAS	WHEY	PRO	CAS	WHEY	PRO
Initial body weight (g)	478±9	497±6	493±19	508±13	494±15	502±3
Final body weight (g)	516±8 *	541±9 *	541±22 *	536±17 *	519±13 *	527±10 *
Final body length (cm)	26.5±0.2	26.6±0.3	26.5±0.3	26.9±0.3	27.0±0.3	27.0±0.4
Final BMI (g/cm^2^)	0.74±0.02	0.76±0.02	0.76±0.02	0.74±0.02	0.71±0.01	0.73±0.02
*n*	*8*	*8*	*8*	*8*	*8*	*8*

Values are means ± SEM. BMI: body mass index.

* p<0.05 *vs*. prior to treatment.

**Fig 2:**
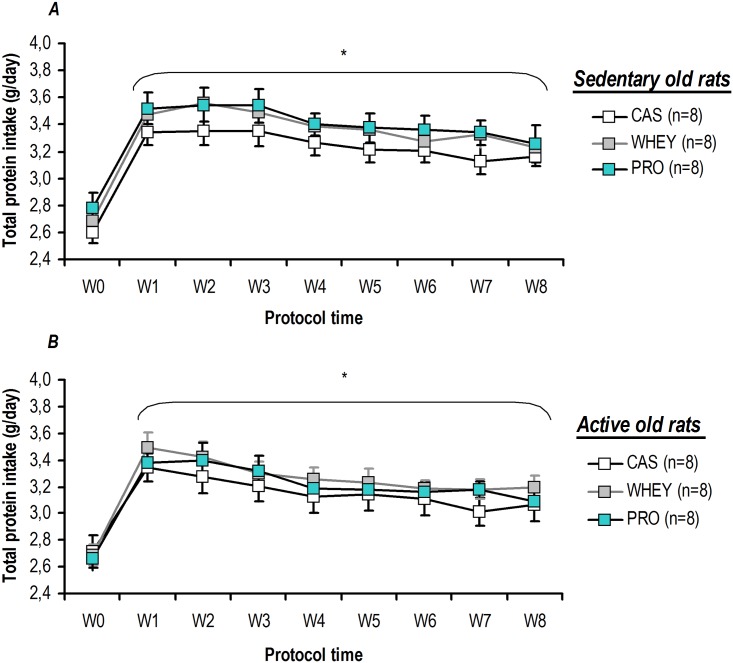
Protein supplementation without food deprivation induced a significant and enduring increase in total protein intake. For sedentary (*A*) and active (*B*) old rats, supplementation significantly increased the total protein intake (*i*.*e* food and specific proteins) over the course of the study period. For each analysed time point, there was an equal amount of total protein ingested, regardless of the specific protein being tested. Values are means ± SEM. W0 to W8: weeks of protocol with W0 being the week prior to physical activity and/or protein supplementation. * p<0.05 *vs*. W0.

**Fig 3:**
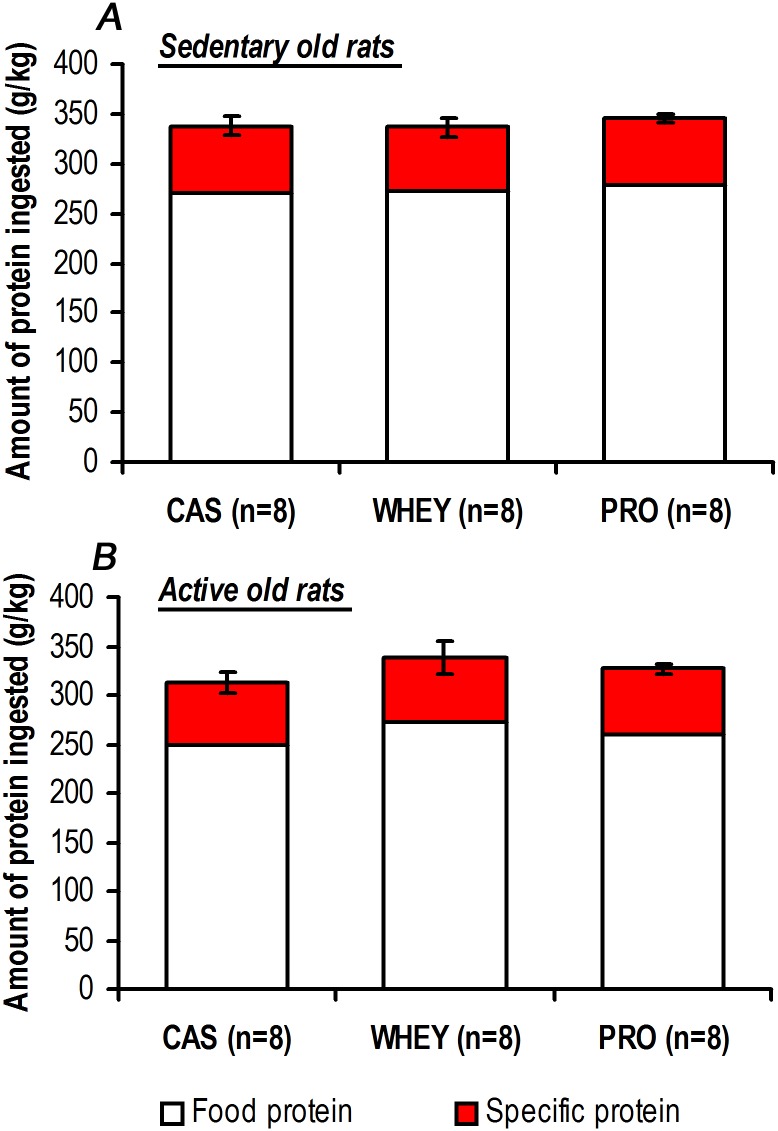
Similar total amount of protein ingested during study in sedentary or active old rats. For each sedentary (*A*) and active (*B*) rats, the weekly protein intake was calculated, normalized to body weight and summarized to compare the total amount of protein ingested during the 2 months of the supplementation with casein, whey or soluble milk protein. With or without a low physical activity, the total amount of protein intake during the 2 months was not significantly different between specific proteins. Values are means ± SEM.

With a similar initial food intake at 17 months of age, all of the groups exhibited a small decrease of this parameter during the study. For all rats, a significant decrease was observed the last two weeks of the treatment (Table 4). This decline was independent of the physical activity or supplementation conditions, since we observed that active and sedentary old rats exhibited the same change between 17 to 19 months of age without dietary supplementation (*i*.*e*. the food intake for sedentary old rats without dietary supplementation went from 25.8±1.1 g/day to 23.3±1.0 g/day, n = 8, p = 0.008; while for active old rats without dietary supplementation it went from 26.3±0.7 g/day to 23.6±1.0 g/day, n = 8, p = 0.016). As illustrated in the Fig 2, administration of a daily bolus of 0.85 g proteins induced a significant increase in the total protein (*i*.*e* food and specific proteins) intake by day for all the rats in active and sedentary conditions. During protein supplementation, the total protein intake was significantly higher than the initial value (W0, *i*.*e*. the week prior to supplementation), despite the decrease in food consumption. No significant difference was found for sedentary or active rats, regardless of the tested protein or the duration of the protocol (Fig 2). Furthermore, the Fig 3 shows that the total amount of proteins (*i*.*e* food and specific proteins) ingested during the 2-months of the study was not significantly different between casein, whey and soluble milk protein in sedentary and active old rats.

**Table 4:** Old rats exhibited a decrease in spontaneous daily food intake.

	Sedentary old rats			Active old rats		
Food intake (g/day)	CAS	WHEY	PRO	CAS	WHEY	PRO
W0	26.0±0.8	26.9±1.0	27.8±1.1	27.2±1.2	26.9±0.8	26.6±0.9
W1	24.9±0.9	26.2±0.9	26.7±1.2	25.0±1.0	26.4±1.2	25.3±1.0
W2	25.0±1.0	27.1±1.1	26.9±1.2	24.3±1.2	25.7±1.1	25.4±1.4
W3	25.0±1.1	26.4±1.0	26.9±1.3	23.5±1.1 *	24.5±0.9	24.7±1.1
W4	24.2±1.0	25.3±0.9	25.5±0.8	22.8±1.2 *	24.1±0.8	23.4±0.7 *
W5	23.6±0.9	25.1±0.9	25.3±1.1	22.9±1.2 *	23.8±1.0	23.3±0.6 *
W6	23.5±0.9	24.3±0.9 *	25.1±1.1	22.6±1.2 *	23.4±0.6 *	23.1±0.4 *
W7	22.8±1.0 *	24.8±0.9 *	24.9±0.9 *	21.6±1.0 *	23.3±0.8 *	23.3±0.6 *
W8	23.1±0.7 *	23.8±0.8 *	24.1±1.4 *	22.1±1.2 *	23.5±0.8 *	22.4±0.8 *
*n*	*8*	*8*	*8*	*8*	*8*	*8*

With an identical initial daily food intake, all groups of old rats exhibited a significant decrease of this parameter, without it being influenced by the protein used for supplementation or by the physical activity condition. Values are means ± SEM. W0 to W8: weeks of protocol, with W0 representing the week prior to physical activity and/or protein supplementation.

* p<0.05 *vs*. W0.

### Physical activity associated with soluble milk protein supplementation increases spontaneous locomotor activity without effect on muscular strength

At the end of the exercise protocol, the analysis of the forelimb grip measurements demonstrated that forces were not significantly different for the sedentary or the active groups, regardless of the protein supplementation (Table 5).

**Table 5:** Sedentary or active old rats had similar forelimb grip strengths regardless of the protein supplementation.

	Sedentary old rats			Active old rats		
Grip test parameters	CAS	WHEY	PRO	CAS	WHEY	PRO
Body weight (g)	519±8	544±8	541±23	542±17	521±12	532±10
Absolute grip force (g)	1121.2±71.7	1135.7±89.7	1142.3±85.6	1087.0±86.4	1103.8±64.3	1163.0±80.1
Relative grip force (g/g BW)	2.15±0.11	2.08±0.15	2.11±0.12	2.03±0.19	2.13±0.14	2.19±0.15
*n*	*8*	*8*	*8*	*8*	*8*	*8*

After two months of protein supplementation with or without a concomitant low-intensity exercise program, no statistical difference was seen in terms of the forelimb grip strength. Values are means ± SEM. BW: Body weight.

**Fig 4:**
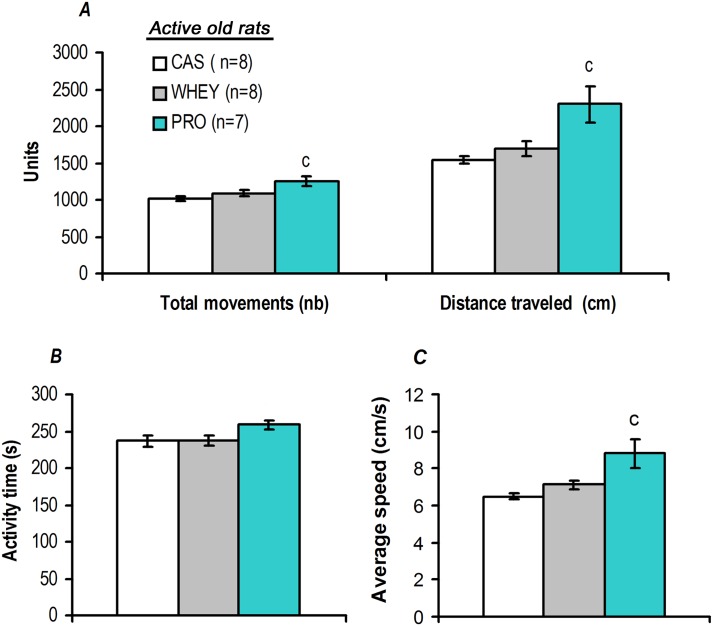
Active old rats supplemented with soluble milk protein exhibited higher locomotion parameters. Assessment of short-term exploration activity using an IR actimeter with 19-month-old rats after two months of protein supplementation with a concomitant low-intensity physical activity program revealed that locomotion capacities were higher for the PRO than for the CAS group. Indeed, active old rats supplemented with soluble milk protein exhibited higher values in terms of the total number of movements and the distance travelled (*A*) and the average speed (*C*), than CAS group. Values are means ± SEM. ^*c*^ p<0.05 *vs*. the CAS group.

As illustrated in Table 6, assessment of short-term exploratory activity demonstrated that locomotor activity was similar for the CAS, WHEY, and PRO sedentary groups. By contrast, physically active old rats supplemented with soluble milk protein PRO exhibited a significant increase in locomotion parameters compared to CAS active old rats (Fig 4). Thus, we found that for the PRO group there was a higher total distance travelled relative to the CAS group of 50% (Fig 4*A*). Activity time was not significantly different for the PRO active group (Fig 4*B*) while calculation of the average speed showed that rodents in PRO active group also exhibited an improvement of this parameter, with a significant increase in their average speed of 35% compared to the CAS group (Fig 4*C*).

**Table 6:** Sedentary old rats exhibited similar locomotion capacities regardless of the protein supplementation.

	Sedentary old rats		
Open field parameters	CAS	WHEY	PRO
Activity time (s)	243±3	258±6	243±6
Total movements (nb)	1102±40	1250±63	1089±43
Distance travelled (cm)	1719±117	1997±169	1676±93
Average speed (cm/s)	7.1±0.4	7.7±0.5	6.9±0.3
*n*	*8*	*8*	*8*

Assessment of short-term exploration activity using an IR actimeter with sedentary rodents after two months of supplementation revealed that locomotion capacities were similar for the three proteins that were administered. Values are means ± SEM.

### Beneficial effects of physical activity on dynamic and static gait parameters in old rats supplemented with proteins

The GaitLab system permits a substantial number of both dynamic and static gait parameters to be captured, thus allowing comparison of the walking properties and capacities of aged rats during unforced walking prior to and following the 2-month protein supplementation with or without physical activity program. With an identical mean of 3 passages for each animal per day, the average of successful trials (*i*.*e*. a regularity of up to 98%) was similar regardless of the type of protein (*i*.*e*. CAS, WHEY, or PRO) or the condition (*i*.*e*. sedentary or active). Analysis of the fore lag and hind lag (*i*.*e*. the time lags between the two feet of the pars) showed that for all of the rats, the frequency of symmetrical gaits was higher than 85% for each group. In light of this finding, analysis of the spatial and temporal parameters was performed on symmetrical gaits. Furthermore, left and right fore- or hindlimb parameters did not differ significantly (p>0.05 by paired t-test). Measurements from right and left limbs were hence pooled.

To quantify gait parameter properties of sedentary and active rodents that received the protein supplements, trials representing the best performance (*i*.*e*. the maximum voluntary speed) prior to and following the 2-month treatment period were chosen for each animal, and pooled (Fig 1). Statistical analysis of parameters obtained prior to the treatment showed that gait properties obtained from 17-month-old rats were similar for all of the groups (Tables 7 and 8). Thus, the findings essentially amounted to: a maximum spontaneous walking speed of 50.5±1.3 cm/s, (n = 46); an ipsilateral time lag greater than 55% of the stride time, which is a key characteristic of lateral walk [31]; stride lengths of nearly 16 cm; a duty factor greater than 50% of the stride time; and a higher brake time for the forelimbs than for the hindlimbs. Ipsilateral time lags were similar prior to and following treatment, regardless of the protein supplementation or the physical activity condition (Tables 7 and 8), thus showing that the variation of dynamic or static parameters described below could not be associated with a change in the limb coordination patterns.

**Table 7:** Stride frequencies increase in old sedentary rats with soluble milk protein supplementation.

	CAS		WHEY		PRO	
Gait parameters	Before	After	Before	After	Before	After
Speed (cm/s)	50.7±3.9	51.4±3.4	49.7±2.7	51.6±1.6	50.9±2.5	54.5±2.8
Regularity (%)	99.1±0.1	99.0±0.1	99.2±0.1	98.9±0.2	99.1±0.2	99.0±0.2
*Lag (% stride time)*						
FL	49.4±0.7	48.3±0.7	50.3±1.2	50.8±0.9	51.3±1.5	51.6±0.8
HL	50.9±1.2	48.6±1.1	50.1±0.7	48.8±0.9	51.0±1.4	50.8±1.3
Left limb	60.0±2.3	57.6±2.2	55.5±1.7	54.4±1.6	57.6±2.0	53.0±1.5
Rigth limb	58.1±2.7	58.4±2.6	56.0±2.0	56.2±1.3	56.8±2.1	54.1±1.4
*Stride length (cm)*						
FL	16.1±0.7	17.0±0.6	16.1±0.5	16.8±0.5	16.6±0.4	16.8±0.8
HL	16.1±0.7	17.2±0.7	16.0±0.5	16.7±0.4 *	16.4±0.4	16.5±0.6
*Stride time (ms)*						
FL	309.1±14.0	310.3±10.9	308.6±14.0	309.1±12.9	311.8±14.3	291.5±10.5 *
HL	307.8±14.3	311.3±11.1	305.4±13.5	305.2±12.3	308.6±13.5	289.1±10.7 *
*Stride frequency (stride per min)*						
FL	196.8±8.5	195.1±7.2	197.7±10.5	196.4±8.0	194.7±8.1	207.3±7.0 *
HL	197.8±8.8	194.5±7.1	199.6±10.4	198.7±7.6	196.5±7.7	209.2±7.5 *
*Stance time (ms)*						
FL	169.5±12.2	167.0±9.0	167.7±11.9	163.5±8.4	170.8±11.6	154.1±8.0
HL	180.8±11.7	177.7±9.9	178.2±13.7	175.3±10.9	181.0±10.6	166.7±9.8
*Duty factor (% stride time)*						
FL	54.4±1.6	53.6±1.4	53.9±1.8	52.8±0.7	54.5±1.6	52.8±1.8
HL	58.5±1.2	56.8±1.5	57.7±2.3	57.2±1.6	58.5±1.4	57.4±1.6
*Brake time (ms)*						
FL	87.4±7.1	88.4±6.1	95.5±6.9	90.0±5.7	95.6±7.5	88.8±6.6
HL	74.1±11.4	57.8±7.7	53.0±5.4	63.8±5.8	65.6±8.4	50.8±2.3
*Brake time (% stance time)*						
FL	52.4±3.7	53.2±2.8	57.5±2.9	55.4±2.7	55.7±1.6	57.3±2.0
HL	41.1±5.9	32.6±3.8	29.9±2.3	37.2±3.6	36.8±5.2	31.1±2.3
*n*	*8*		*8*		*7*	

Successful and symmetrical trials representing the best performance (*i*.*e*. the maximum voluntary speed) prior to and following treatment were chosen for each sedentary animal, pooled, and compared with a Wilcoxon matched-paired signed rank test (comparison of “After” *vs*. “Before” treatment). The supplementations over the 2-month period did not improve the maximum voluntary travel speeds and the walking pattern (Lag and duty factor) of old sedentary rodents. We observed an improvement of static parameters in WHEY group as illustrated by the significant increase of the hindlimb stride length. Only the old rats in the PRO supplemented group exhibited significant changes in dynamic parameters with an increase in stride frequencies. Values are means ± SEM. FL: forelimbs; HL: hindlimbs.

* p<0.05 *vs*. prior to treatment.

**Table 8:** Dynamic and static gait parameters improved in old active rats supplemented with soluble milk protein.

	CAS		WHEY		PRO	
Gait parameters	Before	After	Before	After	Before	After
Speed (cm/s)	49.1±4.4	55.7±4.1	52.9±2.7	61.3±4.2 *	49.9±2.5	60.7±3.4 *
Regularity (%)	99.0±0.1	98.7±0.3	99.4±0.1	99.1±0.2	99.1±0.2	99.1±0.1
*Lag (% stride time)*						
FL	50.0±0.9	49.6±1.2	50.2±0.8	51.4±0.9	50.2±1.0	49.8±1.3
HL	48.5±1.1	49.0±0.9	49.6±0.8	50.4±1.4	50.5±1.0	51.0±1.1
Left limb	55.2±1.6	54.1±1.3	56.3±2.2	54.3±3.5	55.7±1.5	56.7±1.6
Rigth limb	56.8±1.9	54.8±0.9	56.4±2.3	55.1±3.3	55.6±1.9	55.3±1.6
*Stride length (cm)*						
FL	15.5±0.6	17.5±0.5 *	16.8±0.5	19.0±1.0 *	16.6±0.4	18.2±0.4 *
HL	15.5±0.6	17.1±0.5 *	16.6±0.6	18.4±0.9 *	16.4±0.3	18.0±0.4 *
*Stride time (ms)*						
FL	304.1±20.5	302.3±15.6	303.6±8.5	293.3±9.5	319.4±13.4	291.1±15.1
HL	309.7±23.0	292.7±15.4	300.8±6.4	286.3±8.5	316.3±13.3	285.7±15.6 *
*Stride frequency (stride per min)*						
FL	203.0±12.3	201.9±9.7	198.6±5.7	205.9±6.6	190.2±8.1	209.9±10.5 *
HL	200.5±13.4	208.7±10.2	200.0±4.3	210.7±6.4	192.2±8.4	214.2±11.1 *
*Stance time (ms)*						
FL	170.7±15.9	151.1±10.1	162.0±8.3	145.9±10.2 *	174.6±9.7	144.2±11.2 *
HL	186.4±17.0	162.5±13.9	172.7±6.9	158.0±10.6	188.7±10.1	150.2±12.1 *
*Duty factor (% stride time)*						
FL	55.6±1.9	49.8±1.7	53.2±1.7	49.5±2.3 *	54.5±1.3	49.2±1.8
HL	59.8±1.5	54.9±2.1	57.5±2.1	54.9±2.4	59.5±1.0	52.1±1.8 *
*Brake time (ms)*						
FL	98.7±10.8	77.0±5.3 *	90.4±6.2	77.2±5.9 *	100.6±6.0	82.3±7.5 *
HL	63.6±8.5	54.1±5.6	47.1±6.0	49.5±6.5	55.6±4.7	60.6±5.0
*Brake time (% stance time)*						
FL	57.4±1.8	51.2±2.2	55.8±2.5	52.9±2.1	57.6±1.3	56.8±2.0
HL	34.5±4.3	34.0±3.8	27.1±2.9	31.5±3.5	30.4±3.9	42.6±5.4 *
*n*	*8*		*7*		*8*	

Successful and symmetrical trials representing the best performance (*i*.*e*. maximum voluntary speed) prior to and following treatment were chosen for each active animal, pooled, and compared with a Wilcoxon matched-paired signed rank test (comparison of “After” *vs*. “Before” treatment). After two months of treatment, rodents in the PRO group exhibited longer stride lengths as in the CAS and WHEY groups. They also exhibited an increase in the stride frequencies. Non-parametric Kruskal-Wallis testing did not, however, allow differences between “After” values obtained for each protein supplementation to be discerned. Values are means ± SEM. FL: forelimbs; HL: hindlimbs.

* p<0.05 *vs*. prior to treatment.

For the sedentary condition (Table 7), there was no improvement of gait properties following the two months of CAS supplementation. For WHEY group, only hindlimb stride length was significantly increased. By contrast, the old rats in the PRO group exhibited a significant improvement of dynamic gait parameters with an increase in forelimb and hindlimb stride frequencies, amounting to 6.8±1.7% and 6.7±2.2%, respectively.

After two months of low physical activity, active rodents of CAS group exhibited a longer fore- and hindlimb average stride lengths (CAS: 12.3±2.9%, n = 8), but not sufficient to induce a significant increase of the maximum voluntary walking speed. Old active rats supplemented with the rapidly digestible proteins (WHEY and PRO) exhibited a significant increase in the maximum voluntary walking speed (WHEY: 15.5±4.3%, n = 7; PRO: 22.4±5.7%, n = 8) (Table 8). This improvement was associated with longer fore- and hindlimb average stride lengths (WHEY: 11.5±2.9%; n = 7; PRO: 9.6±2.0%, n = 8). In the same way as for the sedentary condition, the active PRO group exhibited higher frequencies of limb movements, with a significant increase of 10.5±3.9% and 11.5±3.7% for the fore- and hindlimbs, respectively, while CAS and WHEY groups did not.

### Less muscle mass in old sedentary rats supplemented with soluble milk protein

The analysis of organ and muscle weights at the end of the treatment showed similar organ and thoracic muscle weights for the various protein supplementation groups in the sedentary or the exercised condition (Table 9). However, for sedentary old rats, Edc, Edl and soleus muscle weights were significantly lower in PRO group than in the CAS group, which exhibited the most elevated relative weights. When compared to WHEY group, only Edl muscle mass was significantly lower for old rats supplemented with soluble milk protein. For the active old rats, Edc, BB, TA, Edl and soleus muscle weights were not significantly different whatever the specific proteins tested.

**Table 9:** PRO group exhibited lower limb muscle mass for the sedentary but not for the exercised condition.

	Sedentary old rats			Active old rats		
Weight (mg/g BW)	CAS	WHEY	PRO	CAS	WHEY	PRO
*Organs*						
Liver	26.33±0.75	26.50±0.79	25.55±0.58	25.37±0.45	26.16±0.97	27.56±0.91
Kidney	2.43±0.03	2.32±0.10	2.36±0.03	2.34±0.07	2.47±0.08	2.53±0.10
*Thoracic muscles*						
Heart	2.28±0.04	2.33±0.07	2.22±0.06	2.25±0.07	2.38±0.06	2.32±0.07
Diaphragm	2.16±0.09	2.20±0.09	2.13±0.06	2.27±0.12	2.23±0.07	2.16±0.11
*Limb muscles*						
Edc	0.71±0.01	0.66±0.01	0.64±0.02 ^c^	0.67±0.03	0.70±0.02	0.64±0.02
BB	0.69±0.02	0.66±0.02	0.61±0.02	0.63±0.04	0.71±0.03	0.61±0.03
TA	1.70±0.03	1.65±0.04	1.53±0.05	1.67±0.08	1.72±0.07	1.63±0.06
Edl	0.42±0.01	0.42±0.01	0.37±0.01 ^c^^.^^w^	0.41±0.02	0.42±0.02	0.39±0.02
Soleus	0.41±0.02	0.38±0.02	0.32±0.02 ^c^	0.37±0.03	0.38±0.02	0.36±0.01
*n*	*8*	*8*	*8*	*8*	*8*	*8*

After two months of protein supplementation with or without the low-intensity physical activity program and *in vivo* assessment, animals were weighed and then sacrificed to weight specific organs and muscles. Analysis of the relative weights (*i*.*e*. divided by the body weight) showed that organ and thoracic muscle weights were similar regardless of the protein supplementation or the physical activity condition. For sedentary rodents, Edc, Edl and soleus muscles were significantly lighter in the PRO group than in the CAS group, but this difference was not seen when the animals concomitantly engaged in a low physical activity. Values are means ± SEM. Edc: *extensor digitorum carpi*; BB: *biceps brachii*; TA: *Tibialis anterior;* Edl: *extensor digitorum longus*; BW: body weight.

^*c*^ p<0.05 *vs*. the CAS group.

^*w*^ p<0.05 *vs*. the WHEY group.

## Discussion

The present study was designed to compare the impact of specific dairy protein supplementations on muscular function with or without a low-intensity physical activity program in a rodent model of aging. Our study shows that post-exercise consumption of rapidly digestible soluble milk protein, without parallel change in the standard level of food consumption, was more effective than casein or whey protein at improving the physical performance of aged rats. Thus, we have demonstrated that rats supplemented with soluble milk protein in conjunction with physical activity have higher spontaneous locomotor activity, improved dynamic and static gait parameters without increase in grip strength and mass of analysed muscles.

In this study, 0.85 g of specific proteins were administered daily as small free access drinking boluses to 17 to 19 months old rodents that were not subjected to food or drink deprivation. This daily dose given to old rats for protein supplementation allows us to increase the amount of protein consumption as recommended to maintain muscular function in the elderly. Indeed, to help older people (>65 years) maintain and regain lean body mass and function, previous studies recommend an average daily intake of least 1.0 to 1.2 g/kg/day instead of 0.8 g/kg/day in young people [36]. During this 2-month study period, we observed a slight but nonetheless significant decrease in food consumption by the rodents receiving protein supplementation. However, this decrease was similar to untreated rats, and thus could reflect age-related impairments in spontaneous food intake, as previously reported in old mice and rats [37, 38]. The bolus supplementation used in our study allowed the protein intake to be significantly increased in a similar manner for all rats. These observations validated our experimental design to analyze the effect of various protein supplementations in a rodent model of aging. While the rats had free access to food, they were fed with a low-protein standard diet (10% by weight of protein, instead of the normal protein diet at 16%), since previous studies regarding aging have highlighted the ineffectiveness of protein supplementation in subjects who had adequate energy, nutrient, and protein intakes [39–41]. Furthermore, in light of previous reports showing that ingestion of a nutritional supplement immediately after exercise can improve the net protein balance [42, 43], with stimulation of skeletal muscle protein synthesis [44], boluses as free access drinking solution were given immediately following the treadmill sessions. Lastly, since boluses appeared to be particularly palatable to the rats, this supplementation was also used as a reward following the treadmill sessions, to favour positive rather than punitive reinforcement that is normally induced by giving the animals an electric shock. These experimental conditions permitted the assimilation of boluses in a short time period without the animals exhibiting any visible manifestations of stress.

Under sedentary conditions, spontaneous activity and grip performances of the 19-month-old rats were similar, regardless of the protein supplementation. Furthermore, we observed only minor differences between specific proteins when gait parameters were analysed. Higher limb muscle (Edc, Edl, soleus) mass was observed in rodents supplemented with casein compared with rapidly digestible soluble milk proteins. Previous reports have indicated that rapidly digestible and leucine-rich proteins, such as whey and the soluble milk protein used in this study, may be more effective than casein at increasing muscle protein synthesis in old rats [27]. It has indeed been demonstrated that 22-month-old rats given experimental meals containing milk proteins with a high leucine content for 30 days exhibited an increase in postprandial muscle protein synthesis in the gastrocnemius muscle, as compared to administration of other milk proteins [27]. However, this improvement was not associated with an increase in muscle mass, as we observed in the present work. A more recent study has shown that long-term replacement of casein with whey protein had no effect on muscle post-absorptive and postprandial protein synthesis, as well as on muscle mass [45]. This discrepancy between short (*i*.*e*. postprandial) and long-term effects of ingested proteins on muscle metabolism could be explained by differences in the rate of protein digestion, whereby assimilation of rapidly digestible proteins induces a dramatic, albeit brief, increase in plasma amino acids, while slowly digested proteins allow a prolonged plateau of mild hyperaminoacidemia to be reached [46, 47]. Results from this study and from other labs [27, 45] suggest that rather than favouring a single type of protein, supplementation with a mix of rapidly and slowly digested leucine-rich proteins could be a promising strategy to reduce age-related loss of muscle mass. Interestingly, it should be noted that despite this difference in muscle weights, the three groups of sedentary rodents developed identical absolute forepaw grip forces. The ability of milk protein-supplemented rodents with lighter muscles to produce a force similar to the force produced by those with heavier muscles indicates that a distinction needs to be made between muscle mass and muscle quality. The discrepancy between muscle mass and quality, and their respective involvement in age-related impairment of locomotor function has become an important issue in recent years, in light of the observation that the decline of muscle strength and physical function at old age is accelerated relative to the loss of muscle mass [48, 49]. It has also been demonstrated that lean muscle mass is not always directly proportional to muscle strength in the elderly [50–52], and that maintaining or gaining muscle mass does not prevent the age-related decline in muscle force [49]. These observations led to the concept of dynopenia, *i*.*e*. the loss of muscle power or quality, which might play a prominent role in the age-related disabling process [53, 54]. The main objective of protein supplementation is not only to maintain muscle mass or increase skeletal muscle force in old people but mainly to preserve locomotion and mobility. In our study, in sedentary condition, specific protein supplementations did not showed strong difference between slow- and fast-digestible proteins in the modification of gait parameters. However, in soluble milk protein supplemented old rats who have similar force and lower muscle mass than casein or whey treated rats, we observed a slight increase in limb stride frequencies. If muscle mass and strength remain fundamental outcomes to be analysed to evaluate therapies aimed at curtailing the decline of physical performance associated with the aging process, the analysis of functional mobility and gait parameters should therefore be included.

Since the beneficial effects of exercise on age-related impairment have been widely described and demonstrated in the elderly and in rodent models of aging, the main objective of this study was to compare the effectiveness of protein supplementation in conjunction with a low physical exercise on the whole-body performances in old rats. Half of the treatment groups were hence submitted to a daily 30-min session with a treadmill at a speed of 10 m/min. This is considerably lower than the maximum voluntary speed determined prior to the dietary treatment, which amounts to exercise with a walking speed about 30 m/min. A walking session at moderate speed was chosen because it is consistent with the exercise capabilities of the majority of the elderly. It is less likely to cause injury and can be readily achieved since no specific equipment is required. Under these conditions, we demonstrated that 19-month-old rats supplemented over a period of two months with soluble milk protein had improved locomotion capacities compared to supplementation with casein. Thus, total movements, distance travelled and average speed during short-term activity exploration in an open-field were found to be significantly higher in the PRO group than casein supplemented rodents. A significant increase in the stride frequency was only seen in active rodents supplemented with soluble milk protein, while for the other groups the improvement of speed was solely associated with a longer step. This combined increase in stride frequencies and length could explain the large increase in the gait voluntary speed and the average speed in open-field. While a higher frequency of limb movement could be detrimental to aging rats due to a more substantial cardio-respiratory demand, recent studies have demonstrated the beneficial effects of soluble milk protein in delaying muscle failure in the elderly [55], and on the endurance capacity in active men [56]. Further investigation on the effects of soluble milk protein administration on the endurance capacity of old animals seems to be essential to better appreciate and characterize the benefits of this supplementation on locomotion capabilities. In regard to muscle mass and force, we observed no difference between supplemented active groups. The effectiveness of a low physical activity associated with protein supplementation in increasing locomotion has been clearly demonstrated in old rats, while the mechanisms to explain the specific effects of soluble milk protein remain unclear. Our positive findings raise the possibility that increased dietary protein with fast-digestible soluble milk protein just after exercise may prevent mobility disability independently of muscle mass improvement and may act on some other physiological processes involved in walking, possibly related to neural activation of skeletal muscle, properties of tendons, ligaments and bones.

In this study, the use of the GaitLab system that induces limited stress in old rats allowed the analysis of locomotion in a longitudinal aspect. Pre- and post-treatment measurements are essential to investigate nutritional interventions targeting skeletal muscle tissue. In order to strengthen the design of future studies, pre- and post- measurements of all parameters that characterized locomotion and force would be advised. Indeed, consumption of soluble milk protein, in combination with moderate physical activity improves walking capacity and appears to be a promising and readily achievable strategy to improve functional mobility in older rats. Differences observed between soluble milk protein and casein were subtle, they ask the question of the biological significance in humans and need further studies on the mechanisms to explain our findings.
